# Personalized Management of Patients with Chronic Rhinosinusitis with Nasal Polyps in Clinical Practice: A Multidisciplinary Consensus Statement

**DOI:** 10.3390/jpm12050846

**Published:** 2022-05-23

**Authors:** Eugenio De Corso, Maria Beatrice Bilò, Andrea Matucci, Veronica Seccia, Fulvio Braido, Matteo Gelardi, Enrico Heffler, Manuela Latorre, Luca Malvezzi, Girolamo Pelaia, Gianenrico Senna, Paolo Castelnuovo, Giorgio Walter Canonica

**Affiliations:** 1 A. Gemelli University Hospital Foundation IRCCS, Otorhinolaryngology, Catholic University of the Sacred Heart, 00168 Rome, Italy; 2 Department of Clinical and Molecular Sciences, Università Politecnica delle Marche, 60131 Ancona, Italy; m.b.bilo@staff.univpm.it; 3 Allergy Unit, Department of Internal Medicine, University Hospital Ospedali Riuniti, 60126 Ancona, Italy; 4 Immunoallergology Unit, University Hospital Careggi, 50134 Florence, Italy; andrea.matucci@unifi.it; 5 Otolaryngology Audiology and Phoniatric Operative Unit, Department of Surgical, Medical, Molecular Pathology and Critical Care Medicine, Azienda Ospedaliero Universitaria Pisana, University of Pisa, 56124 Pisa, Italy; v.seccia@ao-pisa.toscana.it; 6 Respiratory Unit for Continuity of Care, IRCCS, Ospedale Policlinico San Martino, 16132 Genoa, Italy; fulvio.braido@unige.it; 7 Department of Internal Medicine (DiMI), University of Genova, 16146 Genoa, Italy; 8 Department of Otolaryngology, University Hospital of Foggia, 71100 Foggia, Italy; matteo.gelardi@unifg.it; 9 Personalized Medicine, Asthma and Allergy-IRCCS Humanitas Research Hospital, 20122 Milan, Italy; enrico.heffler@hunimed.eu (E.H.); giorgio_walter.canonica@hunimed.eu (G.W.C.); 10 Department of Biomedical Sciences, Humanitas University, 20072 Milan, Italy; luca.malvezzi@humanitas.it; 11 Department of Medical Specialties, Pulmonary Unit, Hospital of Massa, 54100 Massa, Italy; manuela.latorre@uslnordovest.toscana.it; 12 Department of Otorhinolaryngology and Head and Neck Surgery, IRCSS Humanitas Research Hospital, 20089 Milan, Italy; 13 Department of Health Sciences, University Magna Graecia, 88100 Catanzaro, Italy; pelaia@unicz.it; 14 Department of Allergology, Azienda Ospedaliera Universitaria Integrata Verona, 37126 Verona, Italy; gianenrico.senna@aovr.veneto.it; 15 Department of Biotechnology and Life Sciences, Università degli Studi dell’Insubria, 21100 Varese, Italy; paolo.castelnuovo@uninsubria.it; 16 Centro di Ricerca UPLOAD, Università dell’Insubria, 21100 Varese, Italy; 17 SC Otorinolaringoiatria ASST Sette Laghi, 21100 Varese, Italy

**Keywords:** nasal polyps, chronic rhinosinusitis, biologics, allergy, asthma, non-steroidal anti-inflammatory drugs, hypersensitivity, type 2 inflammation

## Abstract

Chronic rhinosinusitis (CRS) is a sino-nasal chronic inflammatory disease, occurring in 5–15% of the general population. CRS with nasal polyps (CRSwNP) is present in up to 30% of the CRS population. One-third of CRSwNP patients suffer from disease that is uncontrolled by current standards of care. Biologics are an emerging treatment option for patients with severe uncontrolled CRSwNP, but their positioning in the treatment algorithm is under discussion. Effective endotyping of CRSwNP patients who could benefit from biologics treatment is required, as suggested by international guidelines. Other issues affecting management include comorbidities, such as allergy, non-steroidal anti-inflammatory drug–exacerbated respiratory disease, and asthma. Therefore, the choice of treatment in CRSwNP patients depends on many factors. A multidisciplinary approach may improve CRSwNP management in patients with comorbidities, but currently there is no shared management model. We summarize the outcomes of a Delphi process involving a multidisciplinary panel of otolaryngologists, pulmonologists, and allergist-immunologists involved in the management of CRSwNP, who attempted to reach consensus on key statements relating to the diagnosis, endotyping, classification and management (including the place of biologics) of CRSwNP patients.

## 1. Introduction

Chronic rhinosinusitis (CRS) refers to the inflammation of the mucosa of the nose and paranasal sinuses. It is characterized by nasal congestion and/or nasal discharge for ≥12 weeks; facial pain and pressure and impaired olfaction (hyposmia or anosmia) can also be present [1]. CRS is estimated to be present in 5–15% of the population [1,2,3,4]. More specifically, CRS was found in 12.9% of a European general population sample [5] and in 11.9% of a US general population sample [6].

CRS is broadly differentiated into CRS with nasal polyps (CRSwNP) and CRS without nasal polyps (CRSsNP). The diagnosis of CRSwNP is generally based on nasal endoscopy and the presence of symptoms [1,2,3,4], assessed by detailed patient history and examination of the head and neck [2]. Computed tomography (CT) is not routinely performed but may be used in certain circumstances, such as following a period of medical management or in preparation for surgery [2]. Epidemiology data for CRSwNP are limited, but results of patient surveys suggest a prevalence of between 2.1% and 4.3% in Europe [7], around 1.1% in the US and China, and 2.5% in South Korea [7,8,9]. Overall, CRSwNP is estimated to occur in up to 30% of the CRS population [8]. The management of CRSwNP with mild symptoms involves regular intranasal corticosteroids (INCS) and saline irrigation (first-line treatment) [2]; a short course of oral corticosteroids (OCS) can be prescribed for moderate-to-severe disease or when first-line treatment has failed [1,2,3,4].

As pharmacotherapy often fails to treat and control nasal polyps, endoscopic sinus surgery (ESS) is often required, with a conservative approach (removal of all nasal polyps while preserving the sinus mucosa) or, if indicated, a more radical one such as reboot surgery (complete removal of polyps and sinus mucosa) [10]. However, the combination of pharmacotherapy and surgery often does not achieve disease control in the most severe cases [1,11,12]. Possibly due to repeated exposure to OCS and surgery, CRSwNP is associated with greater morbidity compared with CRSsNP [2]. Furthermore, CRSwNP is often accompanied by asthma, allergy, and non-steroidal anti-inflammatory drug (NSAID)–exacerbated respiratory disease (N-ERD) [2,13], which complicates its management. As many as 40% of cases of CRSwNP may be uncontrolled [12] (i.e., have persistent symptoms in the last three months, or the need for long-term antibiotics or systemic steroids in the last month), despite receiving maximal pharmacotherapy and ESS [13]. Uncontrolled CRSwNP continues to be a difficult-to-treat condition that needs a multidisciplinary effort [13].

The coexistence of CRSwNP with asthma, allergy, and N-ERD suggests a common pathophysiology, and has led to the concept of united airway disease [1,14], which can be defined as “*a pathological continuum due to the interaction between upper and lower airways in allergy, asthma, infection and inflammation*” [1]. Thus, biologics used for the treatment of asthma that target key components of type 2 inflammation have been investigated in patients with CRSwNP, with encouraging results [10,15]. Two biologics, dupilumab (an anti-interleukin (IL)-4-α chain receptor antibody) and omalizumab (an anti-immunoglobulin E (IgE) antibody), have recently been granted European approval as add-on therapy with INCS; dupilumab has been approved for adults with severe CRSwNP in whom systemic corticosteroids and/or surgery do not provide adequate disease control [16], and omalizumab has been approved for adults with severe CRSwNP in whom INCS therapy does not provide adequate control [17]. Mepolizumab (an anti-IL-5 antibody) [18] and benralizumab (an anti-IL-5-α chain receptor antibody) [19] are still under investigation, but are expected to be licensed soon. Such targeted therapies may significantly improve outcomes in patients with uncontrolled CRSwNP and may also allow for precision and personalized medicine.

However, the introduction of biologics in the clinical management of CRS poses challenges. Guidance on the selection of patients who are likely to benefit from biologics is lacking, and patient selection requires an understanding of the disease pathophysiology (endotyping) and phenotyping [20,21]. At the same time, the higher cost of biologics compared with other treatments is a barrier to treatment for some patients [9,10,15].

To address issues related to precision medicine in CRSwNP, including the place of biologics, a multidisciplinary panel of Italian experts involved in the care of CRS patients met via video conferences between November 2020 and March 2021 to develop practice-oriented and consensus-based statements to guide clinicians. The following topics were analyzed and discussed, based on the available evidence and the clinical experience of the panel members: diagnostic work-up; endotypes of CRSwNP; definition of disease severity and control; and management and position of biologics. The goal of the multidisciplinary panel was to produce statements to guide clinicians in the management of CRSwNP, from diagnostic work-up to the selection of patients eligible for treatment with biologics. This report presents and discusses these statements.

## 2. Materials and Methods

### 2.1. Design

Prompted by the recent approval of biologics by the European Medicines Agency for severe CRSwNP treatment, a multidisciplinary panel (scientific board) of experts (three pulmonologists, five allergist-immunologists, and five otolaryngologists) involved in the management of CRS patients in Italy convened to address open questions related to the management of CRSwNP. Panel participants were selected based on their clinical experience over the years in rhinology and namely in chronic rhinosinusitis, with the purpose of gaining an insight into optimal management of CRSwNP in clinical practice. The multidisciplinary panel was designed to represent the three specialties most involved in the care pathway of the disease (i.e., pulmonologists, allergist-immunologists, and otolaryngologists) in the same proportion and distribution as in clinical practice in Italy. Therefore, the panel included fewer pulmonologists than allergist-immunologists and otolaryngologists, as patients with CRSwNP are mostly assessed by allergist-immunologists and otolaryngologists and referred to pulmonologists when multidisciplinary assessment with a focus on asthma comorbidity is required.

Statements were produced based on published evidence (when available), the clinical experience of the panel members, and consensus. Delphi techniques to reach consensus were applied between November 2020 and March 2021.

The Delphi methodology is an iterative process used to achieve consensus from different opinions on open questions that are not sufficiently supported by evidence [22]. A series of statements selected by a scientific board and covering the topics of interest are submitted to a survey panel of experts, who are asked to express their agreement or disagreement with each statement. Statements for which consensus has not been reached are reformulated, and the survey is repeated until a predefined level of consensus is reached.

### 2.2. Development of Consensus Statements

**Figure 1:**
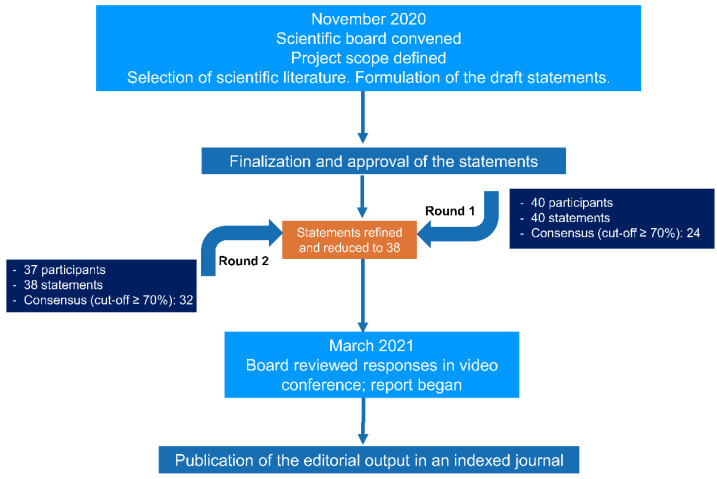
Flow diagram of the Delphi process leading to consensus.

**Figure 2:**
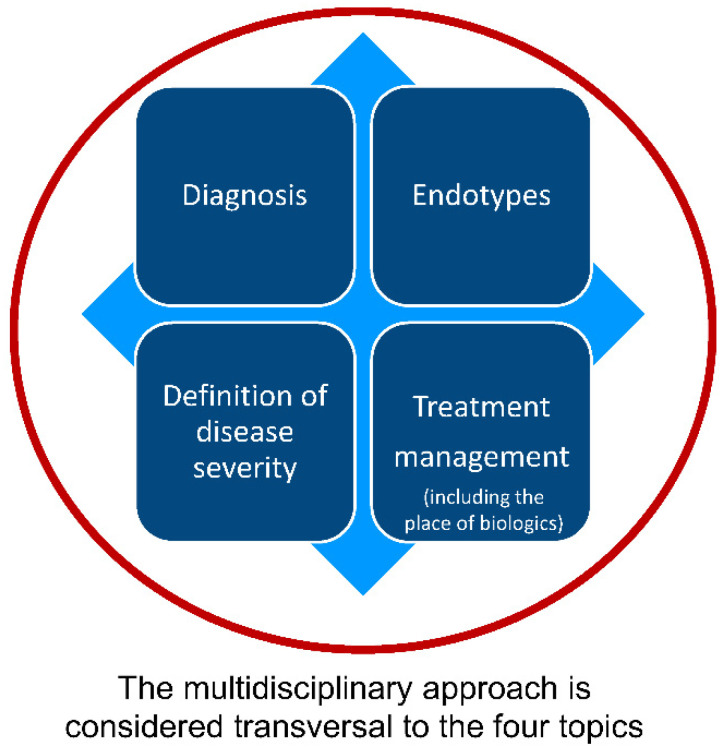
Topics identified as relevant by the scientific board.

A flow chart of the Delphi process used is shown in Figure 1. The scientific board outlined the project scope, designed the strategy for the comprehensive literature search, and selected survey panel members. The board identified the following four topics as relevant: (1) diagnostic work-up; (2) endotypes; (3) definition of disease severity and control; and (4) management of uncontrolled severe CRSwNP, including the place of biologics (Figure 2). The scientific board was then divided into four groups according to the four topics identified, with each group being responsible for the literature search and the production of statements related to the assigned topic. Comprehensive searches of medical databases, including MEDLINE/PubMed, EMBASE, the Cochrane Library, and DB Central, were conducted in December 2020 and repeated in October 2021. Details of the search strategies are provided in Appendix A.

For the selection of the survey panel, each member of the scientific board identified five clinicians known to treat CRS in Italy from the fields of pulmonology, allergy, and otolaryngology to ensure multidisciplinarity. In determining the expertise of the panel members, board members used the following criteria to evaluate the experience of the clinicians: personal and institutional networks, conference presentations, and relevant authorship. 

The first round of Delphi voting took place online using a dedicated platform. The statements were sent to the survey panel members, who were asked to express their agreement/disagreement on each statement using a 5-point Likert scale (1 = totally disagree; 2 = disagree; 3 = partially agree; 4 = agree; and 5 = totally agree). Consensus was defined as ≥70% agreement (i.e., the percentage of votes with scores of 4 or 5). The survey panel participants are listed in Appendix B. 

The first-round responses highlighted several specific topics that had not previously been considered important. In order to ensure that these topics were explored, some statements were updated. Changes between the first and second round included reformulation of the statements where: (1) answers were ambiguous or there was high dispersion of scores; (2) a statement was deemed too generic or was not readily comprehensible; and (3) there was controversy (e.g., a statement contained two different statements, in which case they were divided into separate statements). Where a statement included specific values (e.g., cut-offs), it was opted to include the sources from which they were derived. Finally, to enhance the multiplicity of skills present in the panel, a new question was added asking the specialty of each panelist in order to stratify the answers in the data analysis. 

In March 2021, the results of the second survey were discussed in a plenary video conference attended by the scientific board, and the final version is reported herein.

### 2.3. Ethics

This consensus study was approved by the institutional review boards of all scientific board members who required such authorization. Participation in either round of the Delphi process indicated informed consent on the part of the survey panel. Anonymized results are presented.

### 2.4. Data Analysis 

Consensus on a statement was considered to have been achieved if ≥70% of participants agreed/strongly agreed, or disagreed/strongly disagreed, with it. This is similar to the level of agreement/disagreement considered appropriate in examples of previous Delphi studies [22,23]. Stability of consensus for all relevant items—that is, items that were not modified between rounds—was considered to have been reached when the median response remained ≥4. The results were validated using the “test of the median for independent samples” from SPSS Statistics for Windows, Version 25.0 (IBM Corp., Armonk, NY, USA), which established whether the medians of first and second rounds were the same, using a significance level of 0.05%. Formal statistical testing of differences in responses between panel members from different specialties was not conducted.

## 3. Results and Discussion

Forty of the forty-two clinicians invited to participate in the Delphi survey completed the first round of statement assessments (95% response rate); consensus was reached for 24 of the 40 statements submitted to the survey panel. The list of statements and the percentage agreement for each are shown in Supplementary Table S1 in the Online Resource. In the second round, 37 clinicians (88.1%) reviewed the updated statements. Consensus was reached on 32 of the 38 statements submitted to the survey panel. The 38 statements from the second round are detailed below, grouped according to the four pre-defined themes and key topic areas within each theme. Data on respondents’ discipline were only collected in round 2; there were 17 otolaryngologists, 12 allergists, and 8 pulmonologists. The median scores were similar in both rounds, indicating data stability.

### 3.1. Diagnostic Work-Up (Statements 1–6)

#### 3.1.1. Summary of Statements 

In terms of diagnostic work-up, consensus was reached on five out of six statements (Table 1). Three statements were related to a multidisciplinary approach to diagnosis, and these statements achieved a high level of consensus. Multidisciplinarity allows for overall and early assessment (including atopy and N-ERD), which may prevent the worsening of the disease. Cytology was considered a ‘useful’ tool for cell phenotyping, but not for assessing severity. Smell was considered an element to be investigated, and systematic evaluation with specific tests was considered important, although it did not reach full consensus.

#### 3.1.2. Discussion

Despite the statement on smell assessment (*statement 1*) not reaching consensus, this was still deemed a useful test in patients with CRSwNP symptoms. The lack of consensus may be due to differences between specialists: in general, otolaryngologists are more likely to use smell tests, while pulmonologists and allergists are more likely to use other tools, such as visual analog scales (VAS). However, loss of sense of smell is well understood by all specialists to be a cardinal symptom of CRSwNP [1], and can help confirm CRS and/or nasal polyposis in patients with asthma. Improvement in olfaction is one of the response criteria for biologics in patients with CRSwNP [13], and it was among the efficacy outcomes evaluated in the registration trials of the biologics now approved for CRSwNP [24,25]. The advent of biologics has increased the clinical relevance of this test in patients who are candidates for this treatment. It was therefore agreed that smell assessment be suggested as a recommendation for any specialist who prescribes biologics, while acknowledging they may only be used routinely by otolaryngologists.

Due to the complexity of uncontrolled CRSwNP, a multidisciplinary diagnostic work-up (*statements 2–4*) is mandatory to enable early and targeted interventions [26], as also recommended by current guidelines [13], thus preventing possible disease worsening. In particular, the multidisciplinary team should include a pulmonologist and an allergist, given the high prevalence of concomitant asthma (~50%), atopy (~30%), and N-ERD (10–30%) in patients with CRSwNP [2,27,28,29]. Similarly, patients with moderate-to-severe asthma should be seen by an otolaryngologist to detect CRS and nasal polyposis [27,28,29,30]. 

Atopy should be routinely investigated (*statement 5*) as part of any in-depth diagnostic work-up to detect N-ERD and sensitization to inhalants. Around 10% of patients with CRSwNP also have N-ERD [29], while N-ERD, asthma, and CRSwNP coexist in approximately 16% of patients [31]. There are conflicting data regarding the relationship between atopy and CRSwNP [32]. In some studies, atopy was associated with more severe CRS symptoms [33] or more advanced disease [34], whereas others found little or no correlation between atopy and disease severity [35,36,37]. Patients may also become sensitized to inhalants, which is strongly associated with CRSwNP [29] and the risk of nasal polyps [36], while mucosal inflammation is strongly associated with CRS severity [37]. Nasal cytology *(statement 6*) is used in some otolaryngology centers in Italy but is not as widely used in other countries. Many authors have underlined the importance of local (nasal) markers of inflammation as opposed to systemic markers [38,39,40].

Nasal cytology is easy to perform in the outpatient setting, is inexpensive, and provides information on the cellular components of nasal inflammation, which may help establish whether a patient is a candidate for biologics [26,41,42].

### 3.2. Endotyping (Statements 7–11)

#### 3.2.1. Summary of Statements 

Consensus was reached for four of the five statements relating to endotyping (Table 2). The scientific board agreed on the importance of identifying type 2 inflammation to determine eligibility for biologics and as biomarkers of response. There should be a focus on IgE and eosinophils, despite the lack of consensus regarding the thresholds proposed by the European Position Paper on Rhinosinusitis and Nasal Polyps (EPOS) [1]. IgE plays a key role in CRSwNP, regardless of atopy, and is a strategic target for reducing type 2 inflammation. Endotype-based management of CRS is a relatively recent concept and reflects the approach used in the management of asthma [43,44]. CRS endotyping is useful for understanding the natural course of the disease, making decisions on pharmacotherapy and extent of surgery, and selecting candidates for biologic therapy [44]. Endotyping is highly recommended in patients with severe and recurrent CRSwNP [11], and several approaches have been proposed [20], with the most widely adopted approach to date being based on the presence or absence of type 2 inflammation [45]. Type 2 inflammation is driven by cytokines, including IL-4, IL-5, and IL-13, which regulate immune processes, such as eosinophil recruitment, survival and activation, and IgE synthesis [20]. Elevated serum levels of eosinophils and IgE indicate type 2 inflammation [1,20], which is found in about 80% of CRSwNP cases in Europe and in 95% of cases of moderate to severe CRSwNP [20].

#### 3.2.2. Discussion

There was a high level of consensus among the survey participants regarding the usefulness of eosinophils as a marker of type 2 inflammation (*statement 7*). Eosinophil and IgE cut-off values have only recently been proposed by the EPOS 2020 guidelines [1]; therefore, the lack of consensus on *statement 8* may be due to a lack of awareness of these guidelines, particularly among pulmonologists and allergists. The eosinophil cut-off (≥250 cells/μL) is used to identify patients eligible for biologics, but its presence alone does not define the presence of type 2 inflammation. The same can be said for the IgE cut-off (≥100 kU/L), which is also found in healthy individuals. The lack of consensus may, therefore, also be because these parameters do not always necessarily indicate type 2 inflammation. However, there is a clear need to define eosinophil and IgE cut-offs in order to classify CRSwNP disease severity and identify patients eligible for biologics.

Consensus was reached regarding IgE being a main driver of inflammation *(statement 9*) and a key target for reducing type 2 inflammation (*statement 11*). Components of type 2 inflammation have been implicated in the pathophysiology of CRSwNP, including eosinophilic inflammation and elevated levels of IgE, IL-4, and IL-5, which have long been associated with nasal polyps [45,46,47,48]. This inflammatory pattern, which is driven by preferential activation of T helper 2 cells, leads to increased mucosal inflammation and is common in more severe cases of CRSwNP [48]. Type 2 inflammation is also associated with several coexisting conditions in patients with CRSwNP, including asthma and N-ERD [48]. Elevated nasal tissue IgE and IgE to *Staphylococcus aureus* enterotoxins have been found to correlate with comorbid asthma [20,45,49,50,51,52], and possibly with recurrence of CRSwNP after surgery [53]. Therefore, there is a clear rationale for targeting IgE and other players in the type 2 immune response for the treatment of severe and uncontrolled CRSwNP.

There was consensus on the pathogenic role of IgE in CRSwNP (*statement 10*). However, IgE levels are also believed to be associated with more severe disease. Total IgE levels in nasal polyps are often highly increased independently of atopy and are related to the degree of eosinophilic inflammation [43], while increased local IgE levels suggest comorbid asthma [43]. Thus, the presence of IgE antibodies even in the absence of atopy provides further support for the relationship between type 2 inflammation and disease severity. However, there was disagreement/strong disagreement from 11.1% of panelists regarding the specific serum eosinophil and IgE cut-offs (≥250 cells/μL and ≥100 kU/L, respectively) indicating a type 2 endotype (*statement 8*), although 27.8% were in partial agreement. 

### 3.3. Disease Severity and Control (Statements 12–21)

#### 3.3.1. Summary of Statements

Consensus was reached for nine of the ten statements relating to this topic (Table 3). Assessing disease severity is crucial for therapeutic decision making and for predicting the risk of relapse (uncontrolled disease) [10,54,55]. However, to date, there is no unique definition of disease severity in CRSwNP. These statements aimed to create consensus on some elements for identification of severity, including the nasal polyp score (NPS), the 22-item Sino-Nasal Outcome Test (SNOT-22), use and dose of oral steroids, and cytologic grading. In the EUFOREA 2021 document, uncontrolled CRSwNP is defined as: “persistent or recurring CRSwNP despite long-term INCS, and having received at least one course of systemic corticosteroids in the preceding 2 years and/or previous sinonasal surgery” [10]. Individually, the NPS and SNOT-22 are considered important for assessing the severity of CRSwNP and response to treatment. However, the definition of severity as a function of both NPS and SNOT-22 cut-offs is rapidly evolving. 

In this consensus, it was proposed that the combined use of the two scores provides a more in-depth picture of the disease and a more accurate representation of response to treatment. A score of NPS ≥ 4 is already considered an indication of severity, and it was proposed that this could be a criterion for eligibility for biologics. Level of OCS use was considered useful for assessing disease severity and response to biologics. Presence of N-ERD was also put forward as a criterion for biologic treatment.

#### 3.3.2. Discussion

All statements relating to comorbidities (asthma, non-steroidal anti-inflammatory hypersensitivity, and allergic rhinitis) and the statement relating to nasal cytology separately reached full consensus. However, the statement about the Clinical-Cytological Grading (CCG) (*statement 12*), which includes all these elements, did not reach consensus, possibly due to a general lack of understanding around clinical cytologic grading among pulmonologists and allergists. The CCG evaluates disease severity based on clinical features (in order of increasing severity: presence of hypersensitivity to NSAIDs, asthma, allergy, or NSAID hypersensitivity plus asthma) and cytologic features (in order of increasing severity: tissue neutrophils, mast cells, eosinophils, or eosinophils plus mast cells) to provide a prognostic index of relapse [56,57,58]. Despite being well described in recent years, the CCG is not widely applied in clinical practice. Further research into the use of this scale for classifying severity and assessing prognosis in CRSwNP may allow a greater clinical application of the CCG. 

*Statements 13*, *16*, and *17* reached high consensus levels, confirming the crucial role of NPS and SNOT-22 in measuring CRSwNP severity [59,60,61,62]. Changes in NPS were among the primary efficacy endpoints of the phase 3 trials leading to the approval of dupilumab and omalizumab for CRSwNP, and an NPS cut-off of 5 was used as an eligibility criterion from these studies [24,25], and it has since come into use as a measure of disease severity. Consensus on *statement 13* indicates acceptance of this; however, there was no NPS currently agreed upon as indicating severity. SNOT-22, the only available tool for assessing health-related quality of life (HRQoL) in patients with CRSwNP, was also among the efficacy endpoints of the registration trials [24,25]. There was full consensus that reduction in NPS and SNOT-22 individually adequately describes response to treatment (minimal clinically important difference of SNOT-22: 8.9) (*statements 16 and 17*) [10].

The high level of consensus on *statement 14* reflects the evolution of how disease severity is measured in CRSwNP. However, while EPOS 2020 includes SNOT-22 ≥ 40 as one of the criteria for initiation of biologic treatment (five criteria are required) [1], the EUFOREA 2021 recommendations use a lower SNOT-22 of ≥35 to indicate severe disease [10]. Regardless of the precise cut-off threshold selected, the use of SNOT-22 as an indicator of disease severity means that worse patient-reported outcomes should prompt treatment reassessment. In patients with low baseline SNOT-22 scores, almost all of the score can be attributed to nasal symptoms, while in those with high baseline SNOT-22 scores, nasal symptoms account only for about 50% of the global SNOT-22 value, suggesting that disease pathology has a large impact on emotional well-being and HRQoL [59,62].

Both statements regarding OCS use as a marker of disease severity and treatment response reached consensus (*statements 15 and 19*). Use of OCS provides indirect but useful information on disease severity [61,62,63], while a ≥50% reduction in OCS use is an indirect measure of treatment efficacy, albeit one that is not considered adequate in other diseases (e.g., asthma) [64]. In the EPOS 2020 statement, at least two courses of OCS per year, or long-term (>3 months), low-dose OCS treatment, is one indication for biologics, and a reduction in OCS use is a measure of biologic treatment efficacy [1]. The EUFOREA 2021 document states that there should be no requirement for OCS use following 12 months of biologics [10].

A high level of consensus was reached for *statement 18*, which reflected the most innovative recommendation from EUFOREA 2021 [10]. The combination of SNOT-22 and total NPS in assessing disease severity helps to ensure the patient’s perspective is considered [10], and in the context of the GRADE system of evidence-based medicine this is crucial. The use of both tools in combination indicates a move towards precision medicine for these patients.

CRSwNP and concomitant N-ERD accounts for approximately 10% of people with CRSwNP. This is a difficult-to-treat condition and should be an indication for biologic treatment (*statement 20*), according to the strong consensus of the panel, as well as EUFOREA 2021 [10]. EPOS 2020 does not include N-ERD as a criterion for introducing biologics, but identified the following research priority: “establish whether aspirin desensitization or biologics has better efficacy in patients with N-ERD” [1].

While achieving consensus on the statement regarding the NPS cut-off of ≥4 being a criterion for biologics (*statement 21*), the panel were not unanimous towards lowering the NPS threshold. According to the 2021 EUFOREA paper, severe CRSwNP is indicated by: “bilateral CRSwNP with a NPS of ≥4, and persistent symptoms despite long-term INCS with the need for add-on treatment” [10]; therefore, implying the need for biologics. When considered alongside the high consensus level for *statement 13*, this indicates that our panel considers an NPS ≥ 5 as a more appropriate indicator of disease severity. NPS is not listed among the criteria for biologics in EPOS 2020 [1].

### 3.4. Management of Uncontrolled Severe CRSwNP with Biologics (Statements 22–35)

#### 3.4.1. Summary of Statements

Patients with uncontrolled severe CRSwNP have numerous unmet needs, and biologics represent a valid therapeutic alternative for these patients. A key goal of the consensus process was the identification of eligibility criteria for biologics and markers of treatment response. In order to investigate their most appropriate use, the following issues were identified: (i) the use of biologics in patients never treated by surgery in the presence of predictors of an unfavorable outcome (e.g., elevated NPS to reduce inflammation) and in patients with predictors of poor outcomes with surgery (Table 4); (ii) the supportive role of surgery during treatment with biologics in patients with a moderate response to biologics or in patients with elevated NPS to offer a better starting point (Table 5); and (iii) the use of biologics in difficult-to-treat patients who have undergone multiple surgeries and who have impaired HRQoL regardless of NPS (Table 6). The first seven statements in this section are grouped according to those sub-categories. Next, the statements relating to the evaluation of response to biologics are presented (Table 7). To evaluate the response to biologics, the following criteria are used: reduction in NPS; reduction in SNOT-22 (subject to agreement on cut-off values); improved sense of smell (subject to identification of specific biomarkers of response); and 50% reduction in OCS use.

#### 3.4.2. Use of Biologics in Patients Never Treated by Surgery

The level of agreement for *statement 23* (69.4%) was only marginally below the defined threshold for consensus. This response was low for such an important recommendation which is supported by the evidence and included in the EPOS guidelines [1]. Of note, consensus was reached in the related *statement 24*. A possible explanation why *statement 23* did not reach consensus is that the term “*not eligible*” was too vague to convince the panel. EPOS uses the term “*not fit*” for surgery [1]. Furthermore, this statement specifies only that the CRSwNP be “*serious*” and the term “*uncontrolled*” is missing. This lack of consensus further demonstrates the need for greater clarity in the terminology surrounding CRSwNP to ensure consistent understanding between medical disciplines, which will be important if a multidisciplinary approach is to be applied to this disease.

The panelists agreed that biologics could be started as first-line treatment in some instances—for example, in patients with predictors of poor surgical outcome (asthma, allergy, N-ERD, and elevated markers of type 2 inflammation) (*statement 24*) [31,65,66,67,68,69,70,71,72]. However, there is no universally accepted, standardized method of predicting surgery outcomes. Current guidelines and consensus documents do not include a category for patients who could benefit more from biologics rather than surgery [1,10,73]. Thus, consensus on this statement supports the need for research aimed at identifying patients with a high probability of surgical failure who can therefore be directed to biologics as their first-line therapy (without having surgery), as strongly suggested by the EPOS [1].

*Statement 34* represents another innovative theme on which consensus was reached that would benefit from further research. High levels of preoperative inflammation are associated with poor postoperative disease control [54]. Indeed, up to two-thirds of patients receiving biologics may no longer require surgery after 6–12 months of treatment [10,74]; one might hypothesize that this is a result of reduced inflammation.

#### 3.4.3. Supportive Role of Surgery during Treatment with Biologics

These two statements addressed the potential of concomitant biologics and surgery. In clinical practice, FESS may improve outcomes compared with biologics alone, particularly in patients with very high NPS, while in patients with a moderate response to biologics FESS may help to improve the efficacy of pharmacotherapy. Future studies are needed to confirm these statements.

#### 3.4.4. Use of Biologics in patients who have Undergone Multiple Surgeries

Consensus was reached for both statements regarding the use of biologics in patients treated with multiple surgeries (*statements 25 and 26*). EUFOREA 2019 recommends biologics in patients who have undergone FESS if three of the following criteria are met: type 2 inflammation; two or more courses of OCS in the previous 2 years; significantly impaired QoL; significantly impaired sense of smell; and diagnosis of comorbid asthma [13]. In patients who have not undergone ESS, EUFOREA recommends that four of the criteria mentioned above be met [13]. In patients who have undergone multiple ESS with a significantly impaired QoL, the panel recommended biologics regardless of NPS, which is considered a severity parameter in clinical practice, as multiple surgeries itself is a severity criterion and an important indicator that identifies patients with a predisposition to relapse and those with high levels of type 2 inflammation [45,75].

#### 3.4.5. Evaluation of Response to Biologics

Evidence of type 2 inflammation is one criterion for initiating treatment with the biologics currently available for CRSwNP (*statement 22*) [1,10]. However, strategies that allow more accurate identification of patients eligible for biologics, and specific biomarkers and tools for assessing response to biologics, are currently lacking and are the subjects of intense research [10,54,55,74,76,77,78,79]. What constitutes “clear evidence” of type 2 inflammation is yet to be determined (*see statement 8*). 

*Statement 27*, that biologics should be interrupted if there is no response or poor response after 6 months, achieved a high level of consensus and is a concept supported by EUFOREA 2021 recommendations [10]. However, the EUFOREA 2021 guideline also recommends a trial of ≥12 months if a patient shows response [10]. This further highlights the importance of determining what constitutes a response to biologics to ensure that an adequate treatment trial is conducted. Other open questions include what to do after biologic discontinuation. It may be reasonable to start another biologic with a different type 2 inflammation target. However, to date, there is no experience regarding the order of biologics or the likelihood of response when using a second biologic [10]; this is clearly an unmet need, and one requiring further research.

There was also a high level of consensus for *statement 28* regarding the potential for olfaction recovery with biologics. In phase 3 studies with biologics in patients with CRSwNP, smell significantly improved with dupilumab or omalizumab compared with placebo [24,25]. Therefore, biologics may be a valid alternative to revision surgery for improving olfaction. With respect to smell specifically, more than 60% of people have no improvement after FESS, and around 9% of patients have a worse sense of smell after surgery [80].

*Statement 29* achieved the highest level of consensus in the study. Patient QoL was unanimously recognized as an important treatment outcome. Indeed, there was full agreement that in patients who have undergone multiple ESS with significantly impaired HRQoL, biologics are recommended regardless of NPS (*statement 26*). A reduction in polyp size and improvement in olfaction and QoL all define response to treatment with biologics, according to the consensus panel, ideally using validated cut-offs. According to the EUFOREA group, a satisfactory response is defined by the improvement of at least one symptom or score as follows: improvement of olfaction from anosmia to hyposmia/normosmia; smell score increase of ≥0.5; nasal congestion score decrease by ≥0.5 or objective testing; NPS decrease by 1 by nasal endoscopy; SNOT-22 reduction of ≥8.9; and VAS total symptom reduction of ≥2 cm [10]. 

Otolaryngologists provided the lowest scores for *statement 30*, which did not reach consensus. This reflects the differing scores between specialties, although formal statistical testing was not conducted. Otolaryngologists may not be familiar with biologics in patients with severe uncontrolled CRSwNP, and assume that a lack of response indicates biologic failure rather than a need to switch biologic agents. Greater clarity is needed about the place of biologics in the management of severe uncontrolled CRSwNP, with consistent information communicated to all specialists. Recent recommendations for biologic use in CRSwNP do not stipulate a need for a washout if switching between agents [10,13].

*Statement 31* regarding using the lowest effective dose of systemic corticosteroids achieved high consensus, but panelists were not unanimous. There is a lack of dosing recommendations and thus a wide variation in OCS doses prescribed to patients with CRS (with or without polyps), ranging from 15 mg/day to 1 mg/kg/day (total doses 150–352 mg) [81]. Neither European nor international CRSwNP guidelines provide dosing recommendations for OCS in CRSwNP [1,32,81], indicating a need for further study to establish the optimal dose [81].

*Statement 32* regarding the use of biologics to reduce systemic corticosteroid exposure also had a high level of consensus. Biologics may have a steroid-sparing effect, especially in difficult-to-treat patients such as those with concomitant CRSwNP and asthma [64,82], who often experience more severe symptoms and poor response to treatment. This is an important consideration given the high level of OCS use and associated cost burden in these patients [30], as well as the side effects associated with frequent short courses or long-term use of OCS, including insomnia, mood changes, cardiovascular and gastrointestinal effects, weight gain, and osteoporosis [1,64]. OCS-sparing therapy reduces the daily maintenance dose of OCS required and may also improve certain comorbidities in the context of asthma [64].

### 3.5. Open Questions to Be Addressed in Clinical Trials (Statements 36–38)

The response rates to the issues raised in this survey highlighted areas of unmet need requiring further investigation in clinical trials (*statements 36 and 37*). (Table 8) Predictors of poor control of CRSwNP with standard care need to be defined to aid in decision making regarding whether to perform surgery or to initiate treatment with a biologic. More accurate markers of response to biologics need to be defined; in this regard, nasal cytology may deserve further investigation.

The clinical relevance of nasal IgE to S. aureus endotoxins is yet to be elucidated [49,50,52], but no consensus was reached by the panel as to whether this constituted a key area of future research (*statement 38*).

## 4. Conclusions

This Delphi study has provided further guidance on several areas of the diagnosis and management of CRSwNP for which the narrative is evolving. Newer recommendations are already challenging the traditional concept of nasal polyps as the main differentiator of disease severity in CRS. Guidelines relating to the definitions of, and most appropriate responses to, disease severity and lack of control are changing, as evidenced in the EUFOREA 2021 guidelines [10]. The key is the accurate identification of patients who are most likely to develop uncontrolled disease, and those who are eligible for treatment with biologics. Multidisciplinary management of CRSwNP is needed to identify these patients, including correct diagnosis of comorbidities, such as asthma and N-ERD, which significantly affect disease progression and the treatment pathway. Clearly defined cut-offs for IgE and eosinophils, NPS and SNOT-22, usage and doses of OCS, and use of cytologic grading are all needed to further clarify disease severity. Patients with severe, uncontrolled CRSwNP have numerous unmet needs, and biologics are increasingly becoming an important treatment option in these complex disease scenarios. As such, evaluation of response to biologics will be crucial in terms of reductions in NPS, SNOT-22, and OCS use, and improved sense of smell.

Perhaps the main area for future research should involve defining clinical predictors of poor disease control and how this affects treatment decisions regarding biologics or surgery, and the accuracy of biomarkers (including nasal cytology) for response to biologics. There is a movement towards multimodal precision medicine in all areas of respiratory medicine, and CRSwNP is no exception. 

There is increasing recognition of geographic and ethnic differences in CRSwNP disease characteristics [83]. As such, some of the remarks emerging from this Italian multidisciplinary consensus statement may not be generalizable to the management of CRSwNP in other countries. Nevertheless, we hope that the statements generated in this study will help guide the management of this disease to improve outcomes in these patients. 

## Figures and Tables

**Table 1:** Statements 1–6 from round two of the Delphi process, regarding diagnostic work up, showing response rates among the 37 participants and the level of agreement reached on each statement.

No.	Statements by Topic	Response Rate, n/N (%)	% of Replies ≥ 4 ^a,b^	Strongly Disagree	Disagree	Partially Agree	Agree	Strongly Agree
**1**	In patients with CRSwNP, olfaction should be routinely assessed by means of the University of Pennsylvania Smell Identification Test (UPSIT) or Sniffin’ sticks	37/37 (100.0)	67.6	2.7%	2.7%	27.03%	24.32%	43.24%
**2**	All patients with CRSwNP symptoms should be evaluated in a multidisciplinary fashion to detect the presence of asthma	36/37 (97.3)	**88.9**	2.78%	0.00%	8.33%	33.33%	55.56%
**3**	All patients with moderate/severe asthma should be routinely evaluated by an ear, nose and throat (ENT) specialist to detect the presence of chronic rhinosinusitis and/or nasal polyposis	36/37 (97.3)	**91.7**	2.78%	2.78%	2.78%	25.00%	66.67%
**4**	A multidisciplinary approach enables early detection and management of patients, thus preventing possible worsening of the disease	36/37 (97.3)	**94.4**	2.78%	0.00%	2.78%	36.11%	58.33
**5**	All patients with CRSwNP should be routinely evaluated by a specialist to detect the presence of concomitant atopy with sensitization to aeroallergens and/or drug hypersensitivity	36/37 (97.3)	**94.4**	2.78%	0.00%	2.78%	44.44%	50.00%
**6**	Nasal cytology with sampling of the inferior turbinate is a simple, inexpensive, non-invasive method for the cellular phenotyping of nasal polyposis, and is applicable to outpatient settings	36/37 (97.3)	**75.0**	5.56%	2.78%	16.67%	30.56%	44.44%

^a^ Score 4 = agree and Score 5 = strongly agree (bold values indicates consensus, i.e., ≥70%). ^b^ Stability of consensus (<10% variation) was achieved between round one and round two. Abbreviations: CRSwNP, chronic rhinosinusitis with nasal polyp.

**Table 2:** *Statements 7–11* from round two of the Delphi process, regarding endotyping, showing response rates among the 37 participants and the level of agreement reached on each statement.

No.	Statements by Topic	Response Rate, n/N (%)	% of Replies ≥ 4 ^a,b^	Strongly Disagree	Disagree	Partially Agree	Agree	Strongly Agree
**7**	Values greater than 10 eosinophils per high-powered field (EOS/HPF) in biopsy specimens are indicative of type 2 inflammation	36/37 (97.3)	83.3	2.78%	0.00%	13.89%	72.22%	11.11%
**8**	Eosinophil cut-off point of 250 cells/μL and/or IgE ≥ 100 kU/L, both suggested by EPOS 2020 [1], are indicative of a type 2 endotype	36/37 (97.3)	61.1	2.78%	8.33%	27.78%	44.44%	16.67%
**9**	IgE levels are one of the main drivers of type 2 inflammation in asthma and in CRSwNP	36/37 (97.3)	80.6	0.00%	2.78%	16.67%	52.78%	27.78%
**10**	IgE antibodies play a pathogenic role in CRSwNP, regardless of the patient’s atopic status	36/37 (97.3)	72.2	0.00%	0.00%	27.78%	58.33%	13.89%
**11**	Targeting IgE is a strategy that contributes to reducing type 2 inflammation in CRSwNP	35/37 (94.6)	71.4	0.00%	0.00%	28.57%	54.29%	17.14%

^a^ Score 4 = agree and Score 5 = strongly agree (bold values indicates consensus, i.e., ≥70%). ^b^ Stability of consensus (<10% variation) was achieved between round one and round two. Abbreviations: CRSwNP, chronic rhinosinusitis with nasal polyps; EPOS, European Position Paper on Rhinosinusitis and Nasal Polyps; andIgE, immunoglobulin.

**Table 3:** Statements 12–21 from round two of the Delphi process, regarding disease severity and control, showing response rates among the 37 participants and the level of agreement reached on each statement.

No.	Statements by Topic	Response Rate, n/N (%)	% of Replies ≥ 4 ^a,b^	Strongly Disagree	Disagree	Partially Agree	Agree	Strongly Agree
**12**	In CRSwNP the Clinical-Cytological Grading (CCG) is a useful method for classifying the pathology’s degree of severity	36/37 (97.3)	50.0	5.56%	13.89%	30.56%	36.11%	13.89%
**13**	Total nasal polyp score ≥ 5 can be considered as one of the parameters for CRSwNP severity	36/37 (97.3)	94.4	2.78%	0.00%	2.78%	55.56%	38.89%
**14**	SNOT-22 ≥ 40 (confirmed by EPOS 2020 [1]) is related to CRSwNP severity	36/37 (97.3)	88.9	2.78%	2.78%	5.56%	58.33%	30.56%
**15**	OCS dosage of more than 1 g/year is a sign of CRSwNP severity	36/37 (97.3)	72.2	2.78%	5.56%	19.44%	58.33%	13.89%
**16**	SNOT-22 is the only validated available tool for the assessment of health-related quality of life in CRSwNP patients, and can be considered as a reliable outcome in response to treatment	36/37 (97.3)	77.8	0.00%	0.00%	22.22%	55.56%	22.22%
**17**	Total nasal polyp score reduction can be considered as a reliable outcome in response to treatment	36/37 (97.3)	88.9	2.78%	0.00%	8.33%	61.11%	27.78%
**18**	SNOT-22 and total nasal polyp score are more useful when used together in order to have a deeper insight into the patient’s burden caused by the pathology	36/37 (97.3)	88.9	5.56%	0.00%	5.56%	44.44%	44.44%
**19**	Reduction in systemic prednisone dosage of ≥50% is an indirect outcome in response to biologic treatment	36/37 (97.3)	80.6	2.78%	0.00%	16.67%	61.11%	19.44%
**20**	N-ERD patients are difficult to treat and frequently relapse, and should therefore be considered a candidate to treatment with biologics	36/37 (97.3)	91.7	2.78%	0.00%	5.56%	61.11%	30.56%
**21**	A total nasal polyp score ≥ 4/8, which is one of the criteria for severity suggested by the update of EUFOREA published January 2021 [10], might also be a criterion for eligibility for biologic treatment	35/37 (94.6)	71.4	2.86%	5.71%	20.00%	57.14%	14.29%

^a^ Score 4 = agree and Score 5 = strongly agree (bold values indicates consensus, i.e., ≥70%). ^b^ Stability of consensus (<10% variation) was achieved between round one and round two. Abbreviations: CRSwNP, chronic rhinosinusitis with nasal polyps; EPOS, European Position Paper on Rhinosinusitis and Nasal Polyps; N-ERD, non-steroidal anti-inflammatory drug (NSAID)–exacerbated respiratory disease; OCS, oral corticosteroid; and SNOT, Sino-Nasal Outcome Test.

**Table 4:** Statements 23, 24, and 34 from round two of the Delphi process, regarding use of biologics in patients never treated by surgery, showing response rates among the 37 participants and the level of agreement reached on each statement.

No.	Statements by Topic	Response Rate, n/N (%)	% of Replies ≥ 4 ^a,b^	Strongly Disagree	Disagree	Partially Agree	Agree	Strongly Agree
**23**	Patients with severe CRSwNP not eligible for surgery should be treated with available biologics first line	36/37 (97.3)	69.4	0.00%	11.11%	19.44%	27.78%	41.67%
**24**	Patients with severe CRSwNP may be firstly treated by biologics first-line in the presence of predictors of poor surgical outcome (asthma, allergy, N-ERD, high type 2 biomarkers)	36/37 (97.3)	72.2	0.00%	13.89%	13.89%	30.56%	41.67%
**34**	In patients with high nasal endoscopic polyp scores, treating with biologics before surgery is a driver to reduce the load of inflammation	36/37 (97.3)	72.2	2.78%	11.11%	13.89%	50.00%	22.22%

^a^ Score 4 = agree and Score 5 = strongly agree (bold values indicates consensus, i.e., ≥70%). ^b^ Stability of consensus (<10% variation) was achieved between round one and round two. Abbreviations: CRSwNP, chronic rhinosinusitis with nasal polyps; N-ERD, non-steroidal anti-inflammatory drug (NSAID)–exacerbated respiratory disease.

**Table 5:** Statements 33 and 35 from round two of the Delphi process, regarding the supportive role of surgery during treatment with biologics, showing response rates among the 37 participants and the level of agreement reached on each statement.

No.	Statements by Topic	Response Rate, n/N (%)	% of Replies ≥ 4 ^a,b^	Strongly Disagree	Disagree	Partially Agree	Agree	Strongly Agree
**33**	Functional endoscopic sinus surgery simultaneous to biologic treatment in CRSwNP patients with very high nasal polyps endoscopic scores may offer a better starting point compared with exclusive treatment with biologics	36/37 (97.3)	72.2	2.78%	8.33%	16.67%	41.67%	30.56%
**35**	Functional endoscopic sinus surgery could be a coadjuvant treatment in patients with a moderate response to biologics	36/37 (97.3)	77.8	0.00%	5.56%	16.67%	61.11%	16.67%

^a^ Score 4 = agree and Score 5 = strongly agree (bold values indicates consensus, i.e., ≥70%). ^b^ Stability of consensus (<10% variation) was achieved between round one and round two. Abbreviations: CRSwNP, chronic rhinosinusitis with nasal polyps.

**Table 6:** Statements 25 and 26 from round two of the Delphi process, regarding the use of biologics in patients that have undergone multiple surgeries, showing response rates among the 37 participants and the level of agreement reached on each statement.

No.	Statements by Topic	Response Rate, n/N (%)	% of Replies ≥ 4 ^a,b^	Strongly Disagree	Disagree	Partially Agree	Agree	Strongly Agree
**25**	Treatment with biologics is highly recommended in difficult-to-treat CRSwNP patients who have undergone multiple endoscopic sinus surgeries	36/37 (97.3)	86.1	2.78%	0.00%	11.11%	22.22%	63.89%
**26**	Patients with CRSwNP with a significantly impaired QoL who have undergone multiple appropriate surgery should be eligible for treatment with biologics whatever the nasal polyp score	36/37 (97.3)	72.2	2.78%	8.33%	16.67%	47.22%	25.00%

^a^ Score 4 = agree and Score 5 = strongly agree (bold values indicates consensus, i.e., ≥70%). ^b^ Stability of consensus (<10% variation) was achieved between round one and round two. Abbreviations: CRSwNP, chronic rhinosinusitis with nasal polyps; QoL, quality of life.

**Table 7:** Statements 22, and 27–32 from round two of the Delphi process, regarding the evaluation of response to biologics, showing response rates among the 37 participants and the level of agreement reached on each statement.

No.	Statements by Topic	Response Rate, n/N (%)	% of Replies ≥ 4 ^a,b^	Strongly Disagree	Disagree	Partially Agree	Agree	Strongly Agree
**22**	There should always be clear evidence of type 2 inflammation to consider CRSwNP patients eligible for treatment with available biologics	36/37 (97.3)	91.7	2.78%	0.00%	5.56%	30.56%	61.11%
**27**	Biologics should be discontinued at 6 months of treatment in patients with poor or no response	36/37 (97.3)	86.1	0.00%	0.00%	13.89%	63.89%	22.22%
**28**	Biologics may offer more chance of olfaction recovery compared with revision surgery	36/37 (97.3)	83.3	0.00%	2.78%	13.89%	50.00%	33.33%
**29**	A reduction in polyp size, improvement in sense of smell, and improvement in QoL are criteria to define response to biologics, that should be based on specific cut-offs set by EUFOREA	36/37 (97.3)	97.2	2.78%	0.00%	0.00%	61.11%	36.11%
**30**	In case of discontinuation of a specific biologic, a washout time is not mandatory before starting with another one	36/37 (97.3)	66.7	0.00%	8.33%	25.00%	44.44%	22.22%
**31**	The lowest effective dose of systemic corticosteroids should be used in the short-term management of CRSwNP	36/37 (97.3)	86.1	2.78%	2.78%	8.33%	58.33%	27.78%
**32**	Biologics should be offered for the management of comorbid CRSwNP and asthma in order to reduce exposure to systemic corticosteroids	36/37 (97.3)	88.9	2.78%	0.00%	8.33%	30.56%	58.33%

^a^ Score 4 = agree and Score 5 = strongly agree (bold values indicates consensus, i.e., ≥70%). ^b^ Stability of consensus (<10% variation) was achieved between round one and round two. Abbreviations: CRSwNP, chronic rhinosinusitis with nasal polyps; QoL, quality of life.

**Table 8:** Statements 36–38 from round two of the Delphi process, regarding open questions to be addressed in clinical trials, showing response rates among the 37 participants and the level of agreement reached on each statement.

No.	Statements by Topic	Response Rate, n/N (%)	% of Replies ≥ 4 ^a,b^	Strongly Disagree	Disagree	Partially Agree	Agree	Strongly Agree
**36 ^c^**	Clinical predictors of poor disease control with standard of care (surgery plus local corticosteroids/OCS), to support the decision of whether or not to perform surgery	36/37 (97.3)	83.3	2.78%	2.78%	11.11%	44.44%	38.89%
**37 ^c^**	Accuracy of biomarkers (including nasal cytology) as markers of response to biologics	36/37 (97.3)	91.7	2.78%	0.00%	5.56%	36.11%	55.56%
**38 ^c^**	Clinical usefulness of the detection of Staphylococcus endotoxin-specific IgE at nasal level	36/37 (97.3)	58.3	0.00%	8.33%	33.33%	38.89%	19.44%

^a^ Score 4 = agree and Score 5 = strongly agree (bold values indicates consensus, i.e., ≥70%). ^b^ Stability of consensus (<10% variation) was achieved between round one and round two. ^c^ In response to “Please rate how much a trial addressing this topic would be relevant to advancements in research on CRSwNP”. Abbreviations: IgE, immunoglobulin E; OCS, oral corticosteroid.

## Data Availability

The data presented in this study are available on request from the corresponding author.

## Supplementary Materials

The following supporting information can be downloaded at: https://www.mdpi.com/article/10.3390/jpm12050846/s1, Table S1: Summary of statements from round one of the Delphi process, with level of agreement reached for each.

## Appendix A. Search Strategy

Comprehensive literature searches of medical databases, including MEDLINE/PubMed, EMBASE, the Cochrane Library, and DB Central were conducted in December 2020 and updated in October 2021. The following terms by topic were searched:

*Diagnostic work-up:* ‘nasal cytology’, ‘nasal eosinophilia’, ‘tissue eosinophilia’, ‘multidisciplinary assessment’, ‘comorbid asthma’, ‘early onset asthma’, ‘allergic profile’, ‘endoscopy’, ‘imaging’, ‘quality of life’, and ‘smell’.

*Endotyping*: ‘endotypes’, ‘phenotypes’, ‘IgE’, ‘immunological mechanism’, ‘NSAID-exacerbated respiratory disease’, ‘N-ERD’, ‘eosinophils’, ‘neutrophils’, ‘nasal cytology’, and ‘histology’.

*Disease severity and control:* ‘nasal polyp score’, ‘NPS’, ‘Lund-Mackay score’, ‘severity of disease’, ‘comorbidities’, ‘sino-nasal outcome test’, ‘SNOT-22′, ‘quality of life’, ‘clinical-cytological grading’, and ‘prognostic index of relapse’.

*Management of uncontrolled severe CRSwNP with biologics*: ‘functional endoscopic sinus surgery’, ‘endoscopic sinus surgery’, ‘adequate surgery’, ‘maximal therapy’, ‘topical corticosteroids’, ‘oral corticosteroids’, ‘full functional endoscopic sinus surgery’, ‘radical surgery’, ‘reboot’, ‘side effects’, ‘biomarkers’, ‘blood eosinophilia’, ‘tissue eosinophilia’, ‘omalizumab’, and various drug terms plus ‘nasal polyps’.

## Appendix B. Members of the Panel of Experts and Their Specialty

Diego Bagnasco, University of Genova, Genoa, Italy, *pulmonologist.*

Bianca Beghè, University of Modena and Reggio Emilia, Modena, Italy, *pulmonologist.*

Marco Bonavia, Azienda Sanitaria Locale, ASL 3 Genovese, Genoa, Italy, *pulmonologist.*

Luisa Brussino, University of Torino and Mauriziano Hospital, Turin, Italy, *allergist.*

Mario Bussi, Vita-Salute San Raffaele University, Milan, Italy, *otolaryngologist.*

Marco Caminati, University of Verona, Verona, Italy, *allergist.*

Frank Rikki Canevari, University of Genova, Genoa, Italy, *otolaryngologist.*

Elena Cantone, University “Federico II”, Naples, Italy, *otolaryngologist.*

Elisiana Carpagnano, University of Foggia, Foggia, Italy, *pulmonologist.*

Cristiano Caruso, Fondazione Policlinico Universitario Agostino Gemelli, IRCCS, Rome, Italy, *allergist.*

Michele Cassano, University of Foggia, Foggia, Italy, *otolaryngologist.*

Lorenzo Cecchi, USL Toscana Centro, Prato, Italy, *allergist.*

Nunzio Crimi, University of Catania, Catania, Italy, *allergist and pulmonologist.*

Maria D’Amato, University “Federico II”, Naples, Italy, *pulmonologist.*

Federico Dente, University of Pisa, Pisa, Italy, *pulmonologist.*

Alberto Giulio Dragonetti, ASST Grande Ospedale Metropolitano Niguarda, Milan, Italy, otolaryngologists Andrea Gallo, Sapienza University, Rome, Italy, *otolaryngologist*.

Stefania Gallo, University of Insubria, Varese, Italy, *otolaryngologist.*

Giulia Gramellini, ASST Grande Ospedale Metropolitano Niguarda, Milan, Italy, *otolaryngologist.*

Gabriella Guarnieri, University of Padova, Padova, Italy, *pulmonologist.*

Giuseppe Guida, A.O. S. Croce e Carle, Cuneo, Italy, *allergist.*

Ignazio La Mantia, University of Catania, Catania, Italy, *otolaryngologist*.

Massimo Landi, Azienda Sanitaria Locale ASL 3 Torino, Turin, Italy, *allergist*.

Nicola Lombardo, University Magna Græcia of Catanzaro, Catanzaro, Italy, *otolaryngologist*.

Daniela Lucidi, University of Modena, Modena, Italy, *otolaryngologist*.

Marianna Maffei, A.O.R.N. Ospedali dei Colli, Naples, Italy, *otolaryngologist.*

Giuseppina Manzotti, Casa di Cura Palazzolo, Bergamo, Italy, *allergist*.

Simonetta Masieri, Sapienza University, Rome, Italy, *allergist*.

Francesco Murzilli, Ospedale S.S. Filippo e Nicola, Avezzano (AQ), Italy, *allergist*.

Antonino Musarra, National Health Service, Scilla, Italy, *allergist*.

Giancarlo Ottaviano, University of Padova, Padua, Italy, *otolaryngologist.*

Fabio Pagella, University of Pavia, Pavia, Italy, *otolaryngologist*.

Ernesto Pasquini, AUSL Bologna, Bologna, Italy, *otolaryngologist*.

Vincenzo Patella, Santa Maria della Speranza Hospital, Salerno, Italy, *allergist*.

Carlotta Pipolo, Università degli Studi di Milano, Milan, Italy, *otolaryngologist.*

Ilaria Puxeddu, University of Pisa, Pisa, Italy, *allergist*.

Nicola Quaranta, University of Bari “Aldo Moro”, Bari, Italy, *otolaryngologist.*

Jan Walter Schroeder, ASST Grande Ospedale Metropolitano Niguarda, Milan, Italy, *allergist*.

Nicola Scichilone, University of Palermo, Palermo, Italy, *pulmonologist*.

Matteo Trimarchi, Vita-Salute San Raffaele University, Milan, Italy, *otolaryngologist.*

Giuseppe Valenti, Provincial Outpatient Center of Palermo, Palermo, Italy, *allergist*.
