# Supplementary material for: Personalized Management of Patients with Chronic Rhinosinusitis with Nasal Polyps in Clinical Practice: A Multidisciplinary Consensus Statement
Source: J Pers Med. 2022 May 23;12(5):846. doi: 10.3390/jpm12050846 (PMC9143504; doi:10.3390/jpm12050846)
Supplement: Supplementary file 1 [file jpm-12-00846-s001.zip › jpm-1704537-supplementary.pdf]

Supplementary Material

Table S1. Summary of statements from round one of the Delphi process and the level of agreement reached for each

No.	Statements by topic	% of replies ≥4[a,b]	Strongly disagree	Disagree	Partially agree	Agree	Strongly agree
1	Nasal cytology is an effective tool to determine nasal eosinophilia in CRSwNP.	77.5	0.00%	0.00%	22.50%	35.00%	42.50%
2	Nasal cytology can be an effective surrogate for tissue eosinophilia whenever the histopathologic report is not available.	67.5	2.50%	2.50%	27.50%	32.50%	35.00%
3	In patients with CRSwNP, smell assessment should be routinely assessed by means of UPSIT® test or Sniffin' sticks.	82.5	0.00%	2.50%	15.00%	45.00%	37.50%
4	All patients with CRSwNP with respiratory symptoms must be evaluated to detect the presence of asthma.	100.0	0.00%	0.00%	0.00%	12.50%	87.50%
5	CT imaging should be performed in patients with asthma reporting nasal symptoms.	55.0	0.00%	15.00%	30.00%	20.00%	35.00%
6	In the absence of a biopsy finding, nasal cytology may be helpful in the definition of the phenotype.	75.0	0.00%	2.50%	22.50%	37.50%	37.50%
7	Nasal cytology with sampling on the inferior turbinate is a simple, inexpensive, non-invasive method and also applicable to outpatient settings, for the cellular phenotyping of nasal polyposis.	80.0	0.00%	7.50%	12.50%	32.50%	47.50%
8	Values >10 EOS/HPF in biopsy specimens are indicative of eosinophilic/type 2 involvement.	77.5	0.00%	2.50%	20.00%	62.50%	15.00%

**9**	Eosinophil cut-off point of 250 cells/μL is indicative of a type 2 endotype.	62.5	2.50%	5.00%	30.00%	45.00%	17.50%
**10**	The detection of *Staphylococcus* endotoxin-specific IgE at nasal level is clinically useful.	50.0	0.00%	10.00%	40.00%	37.50%	12.50%
**11**	Targeting IgE is a mechanism to reduce eosinophilic inflammation in asthma and CRS.	60.0	0.00%	12.50%	27.50%	50.00%	10.00%
**12**	In CRSwNP, Clinical-Cytological Grading (CCG) is a simple and fast method to recognize the pathology's degree of severity.	62.5	5.00%	7.50%	25.00%	47.50%	15.00%
**13**	In CRSwNP of moderate degree (CCG 4–6), OCS equal to 800 mg/year is considered an acceptable dose.	40.0	2.50%	20.00%	37.50%	40.00%	0.00%
**14**	Total NPS ≥5 can be considered as a "relative" parameter for the CRSwNP severity.	72.5	0.00%	0.00%	27.50%	65.00%	7.50%
**15**	SNOT-22 ≥40 is related to the disease's severity, but the test is considered to be vulnerable because of its subjectiveness.	47.5	2.50%	7.50%	42.50%	37.50%	10.00%
**16**	OCS dosage >1 g/year is a sign of CRSwNP severity.	85.0	0.00%	5.00%	10.00%	67.50%	17.50%
**17**	Smell must not be considered as the main outcome of treatment since its appearance is usually transitory and brief.	42.5	0.00%	27.50%	30.00%	40.00%	2.50%
**18**	SNOT-22 can be considered a reliable outcome in response to treatment.	90.0	0.00%	2.50%	7.50%	77.50%	12.50%
**19**	Total NPS reduction can be considered a reliable outcome in response to treatment.	80.0	0.00%	2.50%	17.50%	62.50%	17.50%

**20**	Reduction in systemic prednisone dosage of ≥50% is an indirect outcome in response to biologic treatment.	80.0	0.00%	0.00%	20.00%	52.50%	27.50%
**21**	N-ERD patients are problematic under the treatment point of view.	71.8	0.00%	2.56%	25.64%	48.72%	23.08%
**22**	Before patients undergo a FESS surgery, it may be helpful to know their CCG and the prognostic relapse index.	85.0	0.00%	7.50%	7.50%	52.50%	32.50%
**23**	NPS must be adopted on a routine basis.	97.5	0.00%	0.00%	2.50%	55.00%	42.50%
**24**	Up to now, SNOT-22 is the only validated available tool for the assessment of HRQoL in CRSwNP patients.	79.5	0.00%	7.69%	12.82%	56.41%	23.08%
**25**	SNOT-22, CT score and endoscopic score correlate.	67.5	0.00%	10.00%	22.50%	60.00%	7.50%
**26**	A high endoscopic grading score, such as a NPS >5/8 should be added to the criteria of indications for biologic treatment in CRSwNP proposed by EPOS 2020.	67.5	0.00%	10.00%	22.50%	52.50%	15.00%
**27**	There should always be clear evidence of type 2 inflammation to consider CRSwNP patients eligible for treatment with biologics.	92.5	0.00%	2.50%	5.00%	35.00%	57.50%
**28**	Treatment with biologics should be considered in patients never treated by surgery only in exceptional circumstances.	30.0	7.50%	35.00%	27.50%	17.50%	12.50%
**29**	Patients with difficult-to-treat CRSwNP that have undergone multiple appropriate surgeries	57.5	0.00%	22.50%	20.00%	42.50%	15.00%

	should be eligible for treatment with biologics 90.0whatever their endoscopic or HRQoL scores.						
**30**	Biologics should be discontinued at 6 months of treatment in case of poor or no response.	90.0	0.00%	0.00%	10.00%	55.00%	35.00%
**31**	The reduction of polyp size, the improvement of sense of smell and the improvement of QoL are criteria to define response to biologics, that should be based on specific cut-off set by EUFOREA.	94.9	0.00%	0.00%	5.13%	66.67%	28.21%
**32**	In case of discontinuation of a specific biologic, a washout time should be always taken into consideration before starting another one.	50.0	0.00%	20.00%	30.00%	30.00%	20.00%
**33**	The lowest effective dose of systemic CS should be used in the short-term management of CRSwNP.	75.0	0.00%	7.50%	17.50%	50.00%	25.00%
**34**	Biologics should be offered for the management of comorbid CRSwNP and asthma in order to reduce exposure to systemic CS.	95.0	0.00%	0.00%	5.00%	30.00%	65.00%
	Please rate, for each of the following, how much a trial addressing each topic would be relevant to advancements in research on CRSwNP:						
**35**	Efficacy of FESS surgery simultaneous to biologic treatment in severe CRSwNP patients.	67.5	2.50%	7.50%	22.50%	42.50%	25.00%
**36**	Efficacy of treatment with biologics before surgery as a driver to reduction of load of inflammation, in patients with high nasal endoscopic polyp scores.	77.5	0.00%	12.50%	10.00%	47.50%	30.00%

**37**	Comparison between biologics and surgery on recovery of olfaction.	72.5	0.00%	7.50%	20.00%	57.50%	15.00%
**38**	Definition of clinical predictors of poor control of disease with standard of care (surgery plus local corticosteroids/OCS), as a driver of the decision to perform surgery or not.	82.5	0.00%	0.00%	17.50%	65.00%	17.50%
**39**	Efficacy of FESS aimed at improving control of the disease in case of poor or moderate response to the biologics after 6 months of treatment.	65.0	0.00%	5.00%	30.00%	52.50%	12.50%
**40**	Accuracy of biomarkers (including nasal cytology) as markers of response to biologics.	95.0	0.00%	0.00%	5.00%	35.00%	60.00%

Abbreviations: CCG, Clinical-Cytological Grading; CRS, chronic rhinosinusitis; CRSwNP, chronic rhinosinusitis with nasal polyps; CS, corticosteroids; CT, computed tomography; EOS/HPF, eosinophils per high-power field; ESS, endoscopic sinus surgery; FESS, functional endoscopic sinus surgery; HRQoL, health-related quality of life; IgE, immunoglobulin E; OCS, oral corticosteroids; N-ERD, non-steroidal anti-inflammatory drug–exacerbated respiratory disease; NPS, nasal polyp score; OCS, oral corticosteroids; SNOT-22, 22-item sino-nasal outcomes test.
